# Pregabalin Safety in Pregnancy: A Disproportionality Analysis of VigiBase Spontaneous Reporting System

**DOI:** 10.3390/ph18050759

**Published:** 2025-05-20

**Authors:** Sarah Mondada, Francesca Bedussi, Jonathan L. Richardson, Roberta Noseda, Alessandro Ceschi

**Affiliations:** 1 Division of Clinical Pharmacology and Toxicology, Institute of Pharmacological Sciences of Southern Switzerland, Ente Ospedaliero Cantonale, 6900 Lugano, Switzerland; sarah.mondada@eoc.ch (S.M.); francesca.bedussi@eoc.ch (F.B.); alessandro.ceschi@eoc.ch (A.C.); 2 UK Teratology Information Service, Newcastle upon Tyne Hospitals NHS Foundation Trust, Newcastle upon Tyne NE2 4AB, UK; jonathan.richardson3@nhs.net; 3 Clinical Trial Unit, Ente Ospedaliero Cantonale, 6900 Lugano, Switzerland; 4 Faculty of Biomedical Sciences, Università della Svizzera Italiana, 6900 Lugano, Switzerland; 5 Department of Clinical Pharmacology and Toxicology, University Hospital Zurich, 8091 Zurich, Switzerland

**Keywords:** pregabalin, pregnancy, pharmacovigilance, VigiBase, disproportionality, congenital anomalies

## Abstract

**Background/Objectives**: Some product information on pregabalin suggests a potential risk for congenital anomalies (CAs), although evidence remains inconsistent and lacks clear patterns. This study aimed to provide additional safety information on pregabalin use in pregnancy by analyzing VigiBase, the World Health Organization’s global pharmacovigilance database of individual case safety reports (ICSRs). **Methods**: The analysis included de-duplicated ICSRs related to pregabalin exposure in pregnancy, collected up to 16 January 2024. Reporting odds ratios (RORs) with 95% confidence intervals (CIs) were calculated for CA categories reported in at least 10 ICSRs, with a statistical threshold defined as a 95% CI lower bound > 1. **Results**: Among 410 ICSRs, the majority originated from Europe (64.8%) and North America (26.5%). Of these, 59 (14.4%) ICSRs documented only pregabalin exposure in pregnancy, while 351 (85.6%) also reported adverse events in pregnancy. CAs occurred in 82 ICSRs (23.3%), most commonly involving heart defects (30), nervous system anomalies (18), and limb anomalies (12). No signals of disproportionate reporting were identified for these categories compared to the full database (heart defects: ROR 0.587, 95% CI 0.410–0.839; nervous system anomalies: ROR 0.588, 95% CI 0.370–0.933; limb anomalies: ROR 0.671, 95% CI 0.381–1.183). **Conclusions**: Future disproportionality analyses, along with pharmacovigilance and pharmacoepidemiological studies using patient registries and large-scale collaborative projects, are essential for the ongoing monitoring of pregabalin safety in pregnancy.

## 1. Introduction

Pregabalin, a lipophilic analogue of the gamma-aminobutyric acid (GABA) neurotransmitter, has seen increased prescription rates in Europe since 2010 among women of childbearing age, primarily as an adjunct treatment for epilepsy, generalized anxiety disorder, and neuropathic pain [1,2,3,4]. Consequently, assessing the safety of pregabalin use during pregnancy is critical, particularly given concerns about potential adverse outcomes such as small-for-gestational-age (SGA) infants, preterm delivery, and major congenital malformations [5,6]. The guidance on congenital anomalies (CAs), in particular, has been inconsistent: previous observational studies on national healthcare databases have hypothesized potential risks, yet a clear association between pregabalin and teratogenicity or adverse birth or postnatal neurodevelopmental outcomes has not been definitively established [7,8,9]. Various Summaries of Product Characteristics (SmPCs) for pregabalin indicate a potential risk for CAs of the nervous system, eye, orofacial clefts, urinary, and genital malformations following exposure in the first trimester of pregnancy [9,10,11], whilst a comprehensive critical analysis of the available evidence showed it to be inconsistent, with no clear pattern of specific CAs [8]. This inconsistency highlights the need for more data on the occurrence of CAs with pregabalin exposure in pregnancy to better inform clinical decision-making in this vulnerable patient population. Animal studies on the developmental toxicity of pregabalin following foetal exposure also provide inconsistent results. One study found no teratogenic effects in rats or rabbits, even when pregabalin was administered at high doses [12], while another study reported teratogenic effects in rats at therapeutic doses, including a variety of minor external and internal malformations in fetuses, predominantly affecting the limbs, tail, eyes, and abdomen, with findings such as hemorrhages and poor skeletal ossification [13].

This study aimed to add safety information regarding pregabalin use in human pregnancy by analyzing VigiBase, the World Health Organization (WHO) global pharmacovigilance database of individual case safety reports (ICSRs) in order to establish whether there are any signals of disproportionate reporting (SDRs) for CAs associated with pregabalin compared with the full database.

## 2. Results

### 2.1. Characteristics of the Study Population

**Figure 1:**
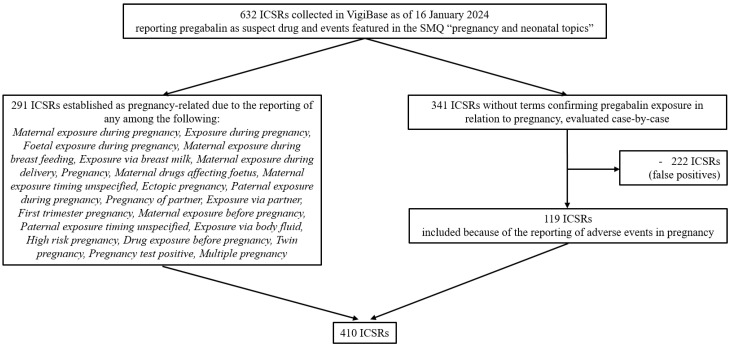
Flowchart summarizing the selection of individual case safety reports included in the study population. Abbreviations: ICSRs, individual case safety reports; SMQ, Standardized MedDRA Query.

As of 16 January 2024, there were over 36 million ICSRs in VigiBase of which 150,405 reported pregabalin as suspect or interacting drug, including 632 mentioning events featured in the standardized MedDRA (Medical Dictionary for Regulatory Activities) query (SMQ) “pregnancy and neonatal topics”. From this initial group of ICSRs, 410 (64.9%) were selected and included in the study population (Figure 1).

The characteristics of the study population are shown in Table 1. The majority of ICSRs were from Europe (266, 64.8%) and were most frequently reported by healthcare professionals (327, 79.8%). A total of 248 (60.5%) ICSRs pertained to patients of female sex while 57 (13.9%) to males (one ICSR involved an adult male who was exposed to pregabalin while the partner was pregnant and who himself reported a type 1 lepra reaction, and the remaining 56 ICSRs referred to a male fetus or neonate/child/infant). A total of 158 ICSRs concerned adults with a median age of 33 years (IQR 28–38 years, n = 130). In most ICSRs, exposure to pregabalin occurred during pregnancy (248, 60.5%). In 11 ICSRs (2.7%), the father was exposed to pregabalin.

### 2.2. Reported Events

Out of 410 ICSRs, 59 ICSRs (14.4%) reported only pregabalin exposure in relation to pregnancy without additional events, whereas 351 (85.6%) also reported additional events. Of these, 48 (13.7%) ICSRs reported “maternal general adverse events” among which the most frequent (i.e., reported in ≥10% of ICSRs) were “drug abuse” (n = 9), “drug withdrawal syndrome” (n = 9), “anxiety” (n = 6), “pain” (n = 6), “suicide attempt” (n = 6), “drug dependence” (n = 5), “insomnia” (n = 5), “nausea” (n = 5), and “weight increased” (n = 5). One ICSR reported lactation insufficiency as a complication, while 315 (89.7%) ICSRs reported a heterogeneous spectrum of adverse events in pregnancy, coded as MedDRA PTs (Table 2). These encompassed pregnancy complications in 35 (11.1%) ICSRs, such as “abortion induced” (n = 9), “caesarean section” (n = 7), and “premature rupture of membranes” (n = 4); “spontaneous abortion” in 104 (33.0%) ICSRs; fetal outcomes in 25 (7.9%) ICSRs of which “foetal growth restriction” (n = 8) and “foetal death”/“stillbirth” (n = 6) were the most frequently reported; neonatal outcomes in 113 (35.9%) ICSRs with “premature” (n = 33) and “small for dates babies” (n = 24) as the most numerous; and 82 (26.0%) ICSRs of CAs (clustered according to the updated EUROCAT guidelines [14]).

### 2.3. Disproportionality Analyses for Congenital Anomalies

Table S1 shows the full list of CAs according to EUROCAT categories reported with pregabalin in the study population. Out of 82 ICSRs with CAs, 62 ICSRs were related to a single category, while 20 ICSRs reported CAs of more than one category. Disproportionality analyses were performed for the three groups of CAs reported in at least 10 ICSRs, which included congenital heart defects (reported in 30 ICSRs), nervous system CAs (in 18 ICSRs), and limb CAs (in 12 ICSRs) (Table 2). No SDRs were detected for any of the three groups compared with the full database: for congenital heart defects, ROR 0.587, 95% CI 0.410–0.839; for nervous system CAs, ROR 0.588, 95% CI 0.370–0.933; and for limb CAs, ROR 0.671, 95% CI 0.381–1.183. Table 3 provides details on ROR computations.

Disproportionality analyses for the same three categories of CAs reported in ICSRs without co-reported drugs alongside pregabalin could not be performed as there were fewer than 10 ICSRs for each of the three categories of CAs of interest (7 ICSRs reporting congenital heart defects, 2 reporting limb CAs and one reporting nervous system CAs). Table S3 provides a detailed list of ICSRs with CAs associated with pregabalin, along with information on co-reported drugs.

## 3. Discussion

### 3.1. Key Results

This study in VigiBase adds safety information regarding pregabalin use in pregnancy and demonstrates that, to date, there are no SDRs for congenital heart defects, nervous system Cas, and limb CAs with pregabalin compared with the full database.

### 3.2. Congenital Anomalies

Our results, which did not identify SDRs, support the recent literature review and critical appraisal by Richardson et al. [8] of studies investigating first trimester maternal pregabalin use and malformation risk, which highlighted that the available evidence is conflicting and generally of low quality, probably influenced by bias and data confounding, with no clear pattern of specific malformations observed.

Moreover, the present study aligns with the communication from the European Network of Teratology Information Services (ENTIS) Scientific Committee, whereby the last updates to the SmPC of pregabalin in relation to congenital malformations from pregabalin exposure in pregnancy were disputed as being based on an unpublished non-peer-reviewed study [15]. Indeed, this same study, when subsequently published in a peer-reviewed scientific journal, did not claim any associations between prenatal pregabalin exposure and risks of major congenital malformations [16]. Our findings are in line with the existing evidence from preclinical studies on pregabalin exposure during pregnancy, which is overall inconsistent and heterogeneous regarding its teratogenic effects [12,13].

Although we found no SDR for cardiac CAs with pregabalin in VigiBase, congenital heart defects were the most frequently reported CAs. This was an interesting finding since this category of CAs was not among those mentioned in the pregabalin SmPCs (FDA, UK and EMA) [9,10,11]. Although one multicenter, observational prospective cohort study of 164 exposed pregnancies and 656 controls linked pregabalin with birth defects, including cardiac anomalies [17], subsequent larger studies did not confirm this association [7,16]. Furthermore, congenital cardiac anomalies constitute a significant proportion of all major Cas, as shown in the systematic review by Denise van der Linde et al. [18], suggesting that their frequency of spontaneous reporting in VigiBase may reflect their baseline frequency. Moreover, the potential causative role played by co-reported drugs besides pregabalin in the onset of cardiac CAs also warrants consideration. It is noted that only a minority of the cardiac CA ICSRs involved pregabalin as the sole reported drug, which did not allow us to assess the presence of SDRs in this subgroup of ICSRs with pregabalin-only exposure.

### 3.3. Other Reported Events

Beyond CAs, our study included several other adverse events in pregnancy associated with pregabalin, among which were spontaneous abortions, premature births, and SGA infants. Moreover, due to the manner in which we selected pregnancy-pregabalin ICSRs, we were able to observe that there were ICSRs in the study population reporting generic maternal adverse events, especially related to drug abuse or withdrawal. Pregabalin abuse and misuse are well documented in the literature, with an updated systematic review from Evoy et al. [19] identifying 55 studies demonstrating a growing trend of harmful use of gabapentin/pregabalin and a 10-year pharmacoepidemiologic study demonstrating likely inappropriate prescription and use [20].

Lastly, our study also identified ICSRs reporting only exposure to pregabalin in relation to pregnancy without additional events, suggesting awareness among healthcare professionals (who make up 80% of the reporters in our study population) of the potential risks associated with off-label pregabalin use in pregnant women. This awareness may motivate reporting to pharmacovigilance systems.

### 3.4. Strengths and Limitations

The primary strength of this study lies in its use of VigiBase, a comprehensive global pharmacovigilance database, which allows for a broad assessment of drug safety across different countries, over an extended period of time and from diverse patient subpopulations, including pregnant women. Additionally, the use of disproportionality analysis is a well-established method for signal detection in pharmacovigilance. The selection of ICSRs using an SMQ with individual case confirmation through a vigorous case validation process produced a robust composite study sample. Whilst other authors who queried the European spontaneous reporting system, EudraVigilance database, excluded ICSRs of sole exposure, as well as ICSRs reporting healthy pregnancy outcomes (e.g., uncomplicated births) and instances of paternal exposure in their algorithm for ICSR selection related to pregnancy, these cases were included in our study as we found these to be equally informative [21].

However, several limitations must be acknowledged. First, the nature of ICSRs means that the data are subject to reporting biases, with potential underreporting or selective reporting. The completeness of ICSRs also relies on the voluntary reporting from different types of reporters, with the unavoidable issue of partial or missing information. Second, the probability that the suspect reported event is drug-related is not the same in all ICSRs since causality cannot be proven. Third, ICSRs within VigiBase lack precise clinical details, meaning that we could not define the exact gestational period (trimester) during pregnancy when exposure to pregabalin occurred. Fourth, the inability to perform subgroup analysis for ICSRs of CAs without co-reported drugs besides pregabalin (due to the paucity of these ICSRs) limits the specificity of our findings regarding pregabalin exposure alone in pregnancy. Indeed, the majority of ICSRs co-reported other drugs in addition to pregabalin, most frequently nervous system agents such as antiepileptics and antidepressants, which may have contributed as confounding factors to the potential association between pregabalin exposure and the reporting of adverse events in pregnancy. Finally, disproportionality analysis is useful as an initial tool for signal detection, but the results it provides require subsequent corroborative observational studies to establish a causal association between drug exposure and the onset of adverse drug reactions (ADRs). Furthermore, the fact that SDRs were not detected for the analysed CAs cannot be interpreted as an endorsement/assurance of safety; disproportionality measures are interdependent, and SDRs are probabilistic calculations of reporting frequencies that contribute to generating hypotheses but cannot be deemed conclusive. Our findings emphasize the need for continued monitoring, as the calculation of RORs depends on the progressively increasing number of ICSRs in VigiBase.

## 4. Materials and Methods

### 4.1. Study Design

This is a descriptive pharmacovigilance study that employed disproportionality analysis using ICSRs collected in VigiBase to evaluate the reporting of CAs in association with pregabalin.

### 4.2. Data Source

VigiBase is the WHO-Uppsala Monitoring Centre’s (WHO-UMC, Uppsala, Sweden) global pharmacovigilance database, providing over 35 million ICSRs of suspected ADRs processed by pharmacovigilance centres in member countries participating in the WHO Programme for International Drug Monitoring (WHO PIDM, Uppsala, Sweden). Contributing member countries of the WHO PIDM (over 170 in 2022) subsequently gain access to VigiBase with the ability to analyse its content. Multiple studies to date have used disproportionality analysis to search for SDRs in relation to drug safety in pregnancy [22,23,24].

### 4.3. Study Population

De-duplicated ICSRs associated with pregabalin and pregnancy, collected in VigiBase since its inception to 16 January 2024 (date of data extraction), were retrieved. The drug of interest, pregabalin, was identified as an “active ingredient” based on WHODrug [25] and was designated by the reporter as “suspect” of a causal relationship with the reported events. The events of interest related to pregnancy were captured using the SMQ “pregnancy and neonatal topics” (MedDRA version 26.1). MedDRA is a hierarchical dictionary used for coding medical events, organized across five levels from very broad to very specific. The highest level is the System Organ Class (SOC), which groups terms by physiological systems or medical disciplines, while the Preferred Term (PT) level provides a standardized description of a single medical concept. The terms included in the selected SMQ correspond to PTs; therefore, PTs were used in this study to describe adverse events associated with pregabalin exposure during pregnancy and for disproportionality analyses. The initial group of ICSRs was filtered according to the reporting of specific terms confirming pregnancy-related drug exposure (listed in Figure 1). The remaining ICSRs lacking the aforementioned terms were identified based on expert opinion, drawing on the expertise of medical professionals and pharmacists with experience in pharmacovigilance. This process was carried out by two pharmacovigilance specialists (SM and RN) and two clinical pharmacologists (FB and AC), who manually reviewed the ICSRs independently. ICSRs reporting specific adverse events in pregnancy were included in the study population (for the complete list of ICSRs included with this procedure, see Table S2). Figure 1 illustrates the flow chart detailing the selection process of the study population.

### 4.4. Variables

The characteristics described for ICSRs included in the study population encompassed administrative information (country of origin, year of reporting, type of reporter), patient demographics (sex, age group, and age in years), details on pregabalin treatment (indication and exposure time in relation to pregnancy—categorized as “before pregnancy”, “during pregnancy”, “both during pregnancy and lactation”, “during lactation”, and via “paternal exposure”, which is drug transmission via semen), and co-reporting of additional drugs (aside from pregabalin). Co-reported drugs were defined as any additional drugs reported alongside pregabalin in the ICSRs, irrespective of whether the reporter considered them as suspect, interacting, or concomitant in relation to adverse event occurrence.

Reported events were grouped into “generic maternal adverse events”, “adverse events in pregnancy, coded as MedDRA PTs” (including pregnancy complications, spontaneous abortion, fetal outcomes, CAs, and neonatal outcomes), and “complications during lactation”. CAs were clustered according to the updated EUROCAT guidelines [14].

### 4.5. Statistical Analysis

The study population was described in terms of absolute numbers with percentages (if categorical) or as median and interquartile range (IQR, if continuous).

In disproportionality analysis, the reporting frequency of a specific drug-event combination is related to the reporting frequencies of all other drugs and all other events present within the full database used as the reference group [26]. Using a 2 × 2 contingency table, the reporting odds ratio (ROR) and its 95% confidence interval (CI) were calculated to identify SDRs for groups of CAs reported with pregabalin. RORs and 95% CI were only calculated for the CA groups reported in at least 10 ICSRs. This threshold was chosen to minimize the risk of detecting spurious associations, as smaller sample sizes can lead to statistical variability. While common practice in pharmacovigilance often uses thresholds of 3 or 5 ICSRs, we opted for a more stringent threshold to ensure that the signals identified were robust and statistically meaningful [27]. This approach aligns with previous pharmacovigilance studies, which have successfully applied disproportionality analysis at the level of SMQs to detect SDRs [28,29].

The full database was set as the primary comparison group and the statistical threshold whereby ROR was deemed significant was defined when lower bound 95% CI > 1 [22].

To curtail the rate of false positive findings and minimize the risk of signal leakage (i.e., confounding by association occurring when the reported adverse event(s) of interest is/are related to co-administered drug(s), [30,31]), sensitivity subgroup disproportionality analysis for groups of CAs reported in ≥10 ICSRs without co-reported drug(s) beside pregabalin were planned.

Data management and analysis were performed with Microsoft Excel (2010, Microsoft Corporation, Washington, DC, USA) and Statistical Analysis System Software (version 9.4; SA Institute, Cary, NC, USA).

The study was conducted following the REporting of A Disproportionality analysis for drUg Safety signal detection using ICSRs in PharmacoVigilance guidelines (READUS-PV) [32,33].

In accordance with the Swiss Human Research Act (810.30, of 30 September 2011—status as of 1 September 2023, Art.2), from the Federal Assembly of the Swiss Confederation, ethical approval and written informed consent were not required from the Comitato etico cantonale Ticino. Data in VigiBase is anonymized.

## 5. Conclusions

This study showed that, to date, there are no SDRs for heart, nervous system or limb CAs in relation to pregnancies exposed to pregabalin in VigiBase. Taking into account the characteristics of the database we used and the methodology we applied, our results cannot be interpreted as a confirmation of safety. Instead, our results can be seen as complementary to the overall available evidence on the safety of pregabalin in relation to pregnancy. In further support of the ENTIS communication on pregabalin in pregnancy [15], our results indicate that the current information reported in pregabalin SmPCs worldwide about potential malformation risks could be misinterpreted by pregabalin prescribers with potential negative consequences for the health of women who would otherwise require pregabalin for the management of relevant pathological conditions. Future disproportionality analyses will be needed to monitor pregabalin safety in pregnancy in the VigiBase spontaneous reporting system, ideally accompanied by pharmacovigilance and pharmacoepidemiological studies on patient registries and other large-scale collaborative projects.

## Figures and Tables

**Table 1:** Baseline characteristics of the individual case safety reports included in the study population.

Characteristic	n (%), N = 410 ICSRs
Country	
Europe	266 (64.9)
North America	109 (26.6)
Asia	13 (3.2)
Australia	13 (3.2)
South America	5 (1.2)
Africa	4 (0.9)
Reporting year	
2006–2010	52 (12.7)
2011–2015	122 (29.8)
2016–2020	170 (41.5)
2021–2024 (as of 16 January 2024)	66 (16.0)
Reporter	
Physician	210 (51.2)
Other health professional	103 (25.1)
Pharmacist	14 (3.4)
Patient	63 (15.4)
Lawyer	2 (0.5)
Not reported	18 (4.4)
Patient sex	
Female	248 (60.5)
Male	57 (13.9)
Not reported	105 (25.6)
Patient age	
Adult	
Reported	130 (31.7)
Median [IQR], years	33 [28–38]
Not reported (age group: adult)	28 (6.8)
Neonate (<28 days)	78 (19.0)
Infant (≥28 days to ≤1 year)	27 (6.6)
Child (>1 year)	8 (2.0)
Not reported (no age information)	139 (33.9)
Exposure to pregabalin in relation to pregnancy	
Before pregnancy	2 (0.5)
During pregnancy	248 (60.5)
During pregnancy and during lactation	10 (2.4)
During lactation	3 (0.7)
Paternal exposure and transmission of drug via semen	11 (2.7)
Unknown	136 (33.2)
Indication	
Neuropathic pain	65 (15.8)
Other pain disorder	51 (12.4)
Anxiety/Depression	49 (12.0)
Epilepsy	40 (9.8)
Fibromyalgia	27 (6.6)
Not Reported	178 (43.4)
Co-reporting of additional drugs besides pregabalin	281 (68.5)

Abbreviations: ICSR, individual case safety report; IQR, interquartile range.

**Table 2:** Spectrum of adverse events in pregnancy reported in association with pregabalin.

Adverse Events in Pregnancy, Coded as MedDRA PTs	n (%), N = 315 ICSRs
Pregnancy complications	35 (11.1)
- reported in ≥10% of ICSRs:	
Abortion induced	9
Caesarean section	7
Premature rupture of membranes	4
Abortion spontaneous	104 (33.0)
*Foetal outcomes*	25 (7.9)
- reported in ≥10% of ICSRs:	
Foetal growth restriction	8
Foetal death/Stillbirth	6
Congenital anomalies ^§^	82 (26.0)
Congenital heart defects	30
Other neonatal malformations ^Δ^	20
Nervous system congenital anomalies	18
Limb congenital anomalies	12
Genital congenital anomalies	7
Oro-facial clefts	6
Chromosomal disorder	6
Renal congenital anomalies	5
Gastrointestinal congenital anomalies	3
Skeletal congenital anomalies	2
Skin congenital anomalies	2
Respiratory congenital anomalies	2
Urinary congenital anomalies	1
Congenital eye disorder	1
Abdominal wall defects	1
Neonatal outcomes	113 (35.9)
- reported in ≥10% of ICSRs:	
Premature baby	33
Small for dates baby	24
Drug withdrawal syndrome	17
Neonatal respiratory distress	12

^§^ Clustered according to the updated EUROCAT guidelines [14]. ^Δ^ These included: “Congenital anomaly” (11 ICSRs), “Multiple congenital abnormalities” (3 ICSRs), “Foetal malformation” (2 ICSRs), “Congenital diaphragmatic hernia” (2 ICSRs), “Dysmorphism” (1 ICSR), “Otospondylomegaepiphyseal dysplasia” (1 ICSR). Abbreviations: MedDRA, Medical Dictionary for Regulatory Activities; PT, preferred terms; ICSR, individual case safety report.

**Table 3:** Computation of reporting odds ratios and 95% confidence intervals in disproportionality analyses comparing the reporting of categories of congenital anomalies with pregabalin against the full database.

	a	b	c	d	ROR [95% CI]
Heart congenital anomalies	30	150,375	12,378	36,393,041	0.587 [0.410–0.839]
Nervous system congenital anomalies	18	150,387	7414	36,398,005	0.588 [0.370–0.933]
Limb congenital anomalies	12	150,393	4327	36,401,092	0.671 [0.381–1.183]

(a) Number of ICSRs of interest reported with pregabalin. (b) Number of ICSRs reporting events other than those of interest with pregabalin. (c) Number of ICSRs of interest reported within the full database, excluding ICSRs in which pregabalin was listed as a suspect drug. (d) Number of ICSRs reporting events other than those of interest within the full database, excluding ICSRs in which pregabalin was listed as a suspect drug. Abbreviations: ICSR, individual case safety reports; ROR, reporting odds ratio; CI, confidence interval.

## Data Availability

Data is contained within the article and Supplementary Material. Further inquiries can be directed to the corresponding author.

## Supplementary Materials

The following supporting information can be downloaded at https://www.mdpi.com/article/10.3390/ph18050759/s1, Table S1: Congenital anomalies reported with pregabalin in the study population; Table S2: Individual case safety reports included in the study population after case-by-case analysis; Table S3: List of individual case safety reports with congenital anomalies associated with pregabalin, along with information on co-reported drugs.

## Abbreviations

The following abbreviations are used in this manuscript:

CA	Congenital anomaly
ICSR	Individual case safety report
ROR	Reporting odds ratio
CI	Confidence interval
WHO	World Health Organization
MedDRA	Medical Dictionary for Regulatory Activities
SMQ	Standardized MedDRA Query
SDR	signal of disproportionate reporting
ADR	Adverse drug reaction
