# Supplementary material for: Pregabalin Safety in Pregnancy: A Disproportionality Analysis of VigiBase Spontaneous Reporting System
Source: Pharmaceuticals (Basel). 2025 May 20;18(5):759. doi: 10.3390/ph18050759 (PMC12114708; doi:10.3390/ph18050759)
Supplement: Supplementary file 1 [file pharmaceuticals-18-00759-s001.zip › pharmaceuticals-3618494-supplementary.pdf]

**Table S1** Congenital anomalies reported with pregabalin in the study population.

| Category | n (%), N = 82 ICSRs * |
|---|---|
| **Congenital heart defects** | 30 (36.6) |
| Atrial septal defect | 10 |
| Ventricular septal defect | 13 |
| Heart disease congenital | 5 |
| Patent ductus arteriosus | 3 |
| Aorta hypoplasia | 2 |
| Pulmonary artery stenosis congenital | 1 |
| Univentricular heart | 1 |
| Congenital aortic valve stenosis | 1 |
| Coarctation of the aorta | 1 |
| Congenital aortic valve incompetence | 1 |
| Ectopia cordis | 1 |
| Dextrocardia | 1 |
| Double outlet right ventricle | 1 |
| Cardiac aneurysm | 1 |
| **Nervous system congenital anomalies** | 18 (22.0) |
| Microtia | 7 |
| Spina bifida | 3 |
| Hydrocephalus | 2 |
| Cerebral ventricle dilatation | 2 |
| Congenital cerebral cyst | 1 |
| Horner's syndrome | 1 |
| Meningomyelocele | 1 |
| Hydranencephaly | 1 |
| Craniosynostosis | 1 |
| **Limb congenital anomalies** | 12 (14.6) |
| Clinodactyly | 2 |
| Congenital musculoskeletal disorder | 2 |
| Limb reduction defect | 2 |
| Adactyly | 1 |
| Arachnodactyly | 1 |
| Polydactyly | 1 |
| Snydactyly | 1 |
| Congenital musculoskeletal disorder of skull | 1 |
| Congenital foot malformation | 1 |
| Congenital musculoskeletal disorder of limbs | 1 |
| **Genital congenital anomalies** | 7 (8.5) |
| Cryptorchism | 4 |
| Hypospadias | 3 |
| **Oro-facial clefts** | 6 (7.3) |
| Cleft palate | 4 |
| Cleft lip | 3 |
| Cleft lip and palate | 1 |
| Nasopharyngeal atresia | 1 |
| **Chromosomal disorders** | 6 (7.3) |
| Trisomy 21 | 5 |
| Cytogenetic abnormality | 4 |
| Emanuel syndrome | 1 |

| Category | n (%), N = 82 ICSRs * |
|---|---|
| **Renal congenital anomalies** | 5 (6.1) |
| Renal aplasia | 2 |
| Kidney malformation | 1 |
| Congenital renal disorder | 1 |
| Single functional kidney | 1 |
| **Gastrointestinal congenital anomalies** | 3 (3.7) |
| Anterior displaced anus | 1 |
| Pyloric stenosis | 1 |
| Congenital inguinal hernia | 1 |
| **Skeletal congenital anomalies** | 2 (2.4) |
| Hemivertebra cleft | 1 |
| Congenital scoliosis | 1 |
| **Skin congenital anomalies** | 2 (2.4) |
| Naevus flammeus | 1 |
| Telangiectasia congenital | 1 |
| Haemangioma congenital | 1 |
| **Respiratory congenital anomalies** | 2 (2.4) |
| Choanal atresia | 1 |
| Congenital tracheomalacia | 1 |
| **Urinary congenital anomalies** | 1 (1.2) |
| Ureterocele | |
| **Congenital eye disorders** | 1 (1.2) |
| Congenital eye disorder | |
| **Abdominal wall defects** | 1 (1.2) |
| Abdominal wall anomaly | |
| **Other neonatal malformations** | 20 (24.4) |
| Congenital anomaly | 11 |
| Multiple congenital abnormalities. | 3 |
| Foetal malformation | 2 |
| Congenital diaphragmatic hernia | 2 |
| Otospondylomegaepiphyseal dysplasia | 1 |
| Dysmorphism | 1 |

* Out of 82 ICSRs with congenital anomalies, 62 ICSRs related to a single category, while 20 ICSRs reported congenital anomalies in more than one category

*Abbreviations*: ICSR, individual case safety report

**Table S2** Individual case safety reports included in the study population after case-by-case analysis.

| ICSR number | Patient sex | Patient age (years) | Age group | MedDRA preferred term | Category of pregnancy outcome |
|---|---|---|---|---|---|
| 1 | Female | 24 | Adult | Abortion spontaneous | Abortion spontaneous |
| 2 | Male | | Neonate | Foetal growth restriction | Foetal outcome |
| | | | | Placental disorder | Pregnancy complication |
| | | | | Placental infarction | Pregnancy complication |
| 3 | Female | | Neonate | Cleft palate | Congenital anomaly |
| 4 | Female | | | Foetal hypokinesia | Foetal outcome |
| | | | | Foetal death | Foetal outcome |
| 5 | Female | | | Abortion spontaneous | Abortion spontaneous |
| 6 | Male | | Neonate | Congenital anomaly | Congenital anomaly |
| 7 | Female | | | Abortion spontaneous | Abortion spontaneous |
| 8 | Female | 44 | Adult | Abortion spontaneous | Abortion spontaneous |
| 9 | Female | | Infant | Congenital pneumonia | Foetal outcome |
| 10 | Female | | | Premature labour | Pregnancy complication |
| 11 | Male | | Neonate | Neonatal respiratory depression | Neonatal outcome |
| 12 | Female | 47 | Adult | Abortion spontaneous | Abortion spontaneous |
| 13 | Male | | Neonate | Respiratory disorder neonatal | Neonatal outcome |
| | | | | Drug withdrawal syndrome neonatal | Neonatal outcome |
| 14 | Female | | | Abortion spontaneous | Abortion spontaneous |
| 15 | Female | 28 | Adult | Abortion spontaneous | Abortion spontaneous |
| 16 | Female | | Adult | Gestational diabetes | Maternal general adverse outcome |
| 17 | Male | | Neonate | Drug withdrawal syndrome neonatal | Neonatal outcome |
| 18 | Female | | Adult | Abortion spontaneous | Abortion spontaneous |
| 19 | Not reported | | Neonate | Multiple congenital abnormalities | Congenital anomaly |
| | | | | Hydrocephalus | Congenital anomaly |
| | | | | Cleft lip and palate | Congenital anomaly |
| | | | | Double outlet right ventricle | Congenital anomaly |
| | | | | Ureterocele | Congenital anomaly |
| 20 | Female | 41 | Adult | Abortion spontaneous | Abortion spontaneous |
| 21 | Female | 29 | Adult | HELLP syndrome | Pregnancy complication |
| 22 | Female | 26 | Adult | Stillbirth | Foetal outcome |
| 23 | Female | | | Abortion | Abortion spontaneous |
| 24 | Female | 27 | Adult | Abortion spontaneous | Abortion spontaneous |
| 25 | Female | | Adult | Abortion spontaneous | Abortion spontaneous |
| 26 | Female | | Adult | Abortion spontaneous | Abortion spontaneous |
| 27 | Female | | Adult | Abortion spontaneous | Abortion spontaneous |
| 28 | Female | | Adult | Abortion spontaneous | Abortion spontaneous |
| 29 | Female | | Adult | Abortion spontaneous | Abortion spontaneous |
| 30 | Female | | Adult | Abortion spontaneous | Abortion spontaneous |
| 31 | Female | | Adult | Abortion spontaneous | Abortion spontaneous |
| 32 | Female | | Adult | Abortion spontaneous | Abortion spontaneous |
| 33 | Female | | Adult | Abortion spontaneous | Abortion spontaneous |
| 34 | Female | | Adult | Abortion spontaneous | Abortion spontaneous |
| 35 | Female | | Adult | Abortion spontaneous | Abortion spontaneous |

| ICSR number | Patient sex | Patient age (years) | Age group | MedDRA preferred term | Category of pregnancy outcome |
|---|---|---|---|---|---|
| 36 | Female | | Adult | Abortion spontaneous | Abortion spontaneous |
| 37 | Female | | | Abortion spontaneous | Abortion spontaneous |
| 38 | Female | | Adult | Abortion spontaneous | Abortion spontaneous |
| 39 | Female | | Adult | Abortion spontaneous | Abortion spontaneous |
| 40 | Female | | Adult | Abortion spontaneous | Abortion spontaneous |
| 41 | Female | | Adult | Abortion spontaneous | Abortion spontaneous |
| 42 | Female | | Adult | Abortion spontaneous | Abortion spontaneous |
| 43 | Female | | Adult | Abortion spontaneous | Abortion spontaneous |
| 44 | Female | | Adult | Abortion spontaneous | Abortion spontaneous |
| 45 | Female | | Adult | Abortion spontaneous | Abortion spontaneous |
| 46 | Female | | Neonate | Atrial septal defect | Congenital anomaly |
| 47 | Female | 30 | Adult | Foetal death | Foetal outcome |
| | | | | Placental transfusion syndrome | Pregnancy complication |
| 48 | Female | 33 | Adult | Abortion spontaneous | Abortion spontaneous |
| 49 | Female | 37 | Adult | Abortion spontaneous | Abortion spontaneous |
| 50 | Male | | Neonate | Hypoglycaemia neonatal | Neonatal outcome |
| 51 | Male | | Neonate | Agitation neonatal | Neonatal outcome |
| 52 | Male | | Neonate | Kidney malformation | Congenital anomaly |
| 53 | Female | | Adult | Abortion spontaneous | Abortion spontaneous |
| 54 | Female | 33 | Adult | Abortion spontaneous | Abortion spontaneous |
| 55 | Female | 33 | Adult | Abortion spontaneous | Abortion spontaneous |
| 56 | Female | | | Abortion spontaneous | Abortion spontaneous |
| 57 | Female | 41 | Adult | Abortion spontaneous | Abortion spontaneous |
| 58 | Female | 41 | Adult | Abortion spontaneous | Abortion spontaneous |
| 49 | Female | | | Abortion spontaneous | Abortion spontaneous |
| 60 | Male | | Neonate | Hemivertebra | Congenital anomaly |
| 61 | Female | 29 | Adult | Abortion spontaneous | Abortion spontaneous |
| 62 | Female | 36 | Adult | Abortion induced | Abortion induced |
| 63 | Female | 40 | Adult | Abortion induced | Abortion induced |
| 64 | Female | | | Foetal growth restriction | Foetal outcome |
| 65 | Female | | | Hydrocephalus | Foetal outcome |
| | | | | Cerebellar hypoplasia | Foetal outcome |
| | | | | Abortion induced | Abortion induced |
| 66 | Not reported | | | Anembryonic gestation | Pregnancy complication |
| 67 | Female | | Neonate | Congenital musculoskeletal disorder | Congenital anomaly |
| | | | | Atrial septal defect | Congenital anomaly |
| 68 | Female | 23 | Adult | Abortion spontaneous | Abortion spontaneous |
| 69 | Female | 33 | Adult | Abortion spontaneous | Abortion spontaneous |
| 70 | Female | | | Dysmorphism | Congenital anomaly |
| | | | | Clinodactyly | Congenital anomaly |
| | | | | Drug withdrawal syndrome neonatal | Neonatal outcome |
| | | | | Neonatal respiratory distress | Neonatal outcome |
| | | | | Bradycardia neonatal | Neonatal outcome |
| | | | | Hypotonia neonatal | Neonatal outcome |
| | | | | Jaundice neonatal | Neonatal outcome |
| 71 | Female | | Neonate | Congenital anomaly | Congenital anomaly |

| ICSR number | Patient sex | Patient age (years) | Age group | MedDRA preferred term | Category of pregnancy outcome |
|---|---|---|---|---|---|
| 72 | Male | | Neonate | Heart disease congenital | Congenital anomaly |
| 73 | Female | | Neonate | Premature baby | Pregnancy complication |
| | | | | Patent ductus arteriosus | Congenital anomaly |
| | | | | Hydrocephalus | Foetal outcome |
| | | | | Cerebral haemorrhage | Foetal outcome |
| 74 | Male | | Neonate | Univentricular heart | Congenital anomaly |
| | | | | Aorta hypoplasia | Congenital anomaly |
| 75 | Male | | Neonate | Neonatal respiratory distress | Neonatal outcome |
| | | | | Congenital tracheomalacia | Congenital anomaly |
| | | | | Bradycardia neonatal | Neonatal outcome |
| | | | | Hypertonia neonatal | Neonatal outcome |
| | | | | Agitation neonatal | Neonatal outcome |
| 76 | Female | | | Abortion missed | Abortion spontaneous |
| 77 | Male | | Neonate | Premature baby | Pregnancy complication |
| | | | | Hyperbilirubinaemia neonatal | Neonatal outcome |
| | | | | Hypertonia neonatal | Neonatal outcome |
| | | | | Agitation neonatal | Neonatal outcome |
| 78 | Female | 38 | Adult | Abortion spontaneous | Abortion spontaneous |
| 79 | Female | 28 | Adult | Drug withdrawal syndrome neonatal | Neonatal outcome |
| | | | | Neonatal respiratory distress | Neonatal outcome |
| | | | | Atrial septal defect | Congenital anomaly |
| | | | | Patent ductus arteriosus | Congenital anomaly |
| 80 | Female | 29 | Adult | Forceps delivery | Pregnancy complication |
| | | | | Haemorrhage in pregnancy | Pregnancy complication |
| 81 | Female | 40 | Adult | Abortion spontaneous | Abortion spontaneous |
| 82 | Female | 36 | Adult | Abortion spontaneous | Abortion spontaneous |
| 83 | Female | 34 | Adult | Abortion spontaneous | Abortion spontaneous |
| 84 | Female | 30 | Adult | Abortion spontaneous | Abortion spontaneous |
| 85 | Female | 30 | Adult | Caesarean section | Pregnancy complication |
| 86 | Female | 36 | Adult | Abortion spontaneous | Abortion spontaneous |
| 87 | Female | 41 | Adult | Abortion spontaneous | Abortion spontaneous |
| 88 | Female | | | Abortion spontaneous | Abortion spontaneous |
| 89 | Female | | | Premature delivery | Pregnancy complication |
| 90 | Female | 31 | Adult | Abortion spontaneous | Abortion spontaneous |
| 91 | Female | 32 | Adult | Abortion induced | Abortion induced |
| 92 | Female | 39 | Adult | Obstetric procedure complication | Pregnancy complication |
| 93 | Female | | | Abortion spontaneous | Abortion spontaneous |
| 94 | Female | | Adult | Abortion spontaneous | Abortion spontaneous |
| 95 | Female | | | Abortion spontaneous | Abortion spontaneous |
| 96 | Female | 30 | Adult | Hydrops foetalis | Foetal outcome |
| | | | | Premature rupture of membranes | Pregnancy complication |
| 97 | Female | | | Abortion spontaneous | Abortion spontaneous |
| 98 | Male | | Neonate | Heart disease congenital | Congenital anomaly |
| | | | | Polydactyly | Congenital anomaly |
| 99 | Female | 35 | Adult | Abortion spontaneous | Abortion spontaneous |
| 100 | Female | 36 | Adult | Abortion spontaneous | Abortion spontaneous |

| ICSR number | Patient sex | Patient age (years) | Age group | MedDRA preferred term | Category of pregnancy outcome |
|---|---|---|---|---|---|
| 101 | Male | | | Hypoglycaemia neonatal | Neonatal outcome |
| 102 | Male | | Neonate | Hypospadias | Congenital anomaly |
| 103 | Female | 31 | Adult | Abortion | Abortion spontaneous |
| 104 | Female | | | Abortion spontaneous | Abortion spontaneous |
| 105 | Not reported | | | Abortion spontaneous | Abortion spontaneous |
| 106 | Male | | Neonate | Apgar score low | Neonatal outcome |
| 107 | Female | 36 | Adult | Abortion spontaneous | Abortion spontaneous |
| 108 | Female | | | Premature labour | Pregnancy complication |
| 109 | Female | | | Abortion spontaneous | Abortion spontaneous |
| 110 | Female | | | Abortion spontaneous | Abortion spontaneous |
| 111 | Female | | | Abortion spontaneous | Abortion spontaneous |
| 112 | Female | | | Abortion spontaneous | Abortion spontaneous |
| 113 | Female | | | Abortion spontaneous | Abortion spontaneous |
| 114 | Female | 26 | Adult | Abortion spontaneous | Abortion spontaneous |
| 115 | Female | | | Caesarean section | Pregnancy complication |
| 116 | Male | | Neonate | Premature baby | Pregnancy complication |
| | | | | Respiratory disorder | Neonatal outcome |
| | | | | Jaundice | Neonatal outcome |
| | | | | Withdrawal syndrome | Neonatal outcome |
| 117 | Female | 25 | Adult | Caesarean section | Pregnancy complication |
| 118 | Female | 40 | Adult | Foetal disorder | Foetal outcome |
| | | | | Abortion | Abortion spontaneous |
| 119 | Female | 26 | Adult | Abortion | Abortion spontaneous |

*Abbreviations*: ICSR, individual case safety reports; MedDRA, Medical Dictionary for Regulatory Activities

**Table S3** List of individual case safety reports with congenital anomalies associated with pregabalin, along with information on co-reported drugs

| ICSR progressive number | Reported congenital anomaly | Category of congenital anomaly | Co-reported drugs (active ingredients) | Co-reported drugs (ATC codes) |
|---|---|---|---|---|
| 1 | Microtia | Nervous system congenital anomalies | Lamotrigine | N03 |
| 2 | Cleft palate<br>Cleft lip | Oro-facial clefts | Topiramate<br>Fluoxetine hydrochloride<br>Oxycodone<br>Hydrocodone bitartrate;Paracetamol<br>Hydrochlorothiazide;Triamterene<br>Amlodipine besilate;Benazepril hydrochloride<br>Cyclobenzaprine hydrochloride<br>Folic acid | N03<br>N06<br>N02<br>N02<br>C03<br>C09<br>M03<br>B03 |
| 3 | Atrial septal defect<br>Cleft palate<br>Congenital musculoskeletal disorder of skull<br>Cleft lip<br>Craniosynostosis<br>Syndactyly<br>Congenital eye disorder<br>Ventricular septal defect<br>Patent ductus arteriosus | Congenital heart disease<br>Oro-facial clefts<br>Limb congenital anomalies<br>Nervous system congenital anomalies<br>Congenital eye disorder | Topiramate<br>Primidone<br>Quetiapine fumarate | N03<br>N03<br>N05 |
| 4 | Microtia | Nervous system congenital anomalies | Lamotrigine | N03 |
| 5 | Microtia | Nervous system congenital anomalies | Lamotrigine | N03 |
| 6 | Heart disease congenital<br>Polydactyly | Congenital heart disease<br>Limb congenital anomalies | Interferon beta-1a | L03 |
| 7 | Hypospadias | Genital congenital anomalies | Levetiracetam<br>Hydromorphone hydrochloride | N03<br>N02 |

| | | | | |
|---|---|---|---|---|
| 8 | Trisomy 21 | Chromosomal disorder | Gabapentin<br>Metaxalone<br>Hydrocodone bitartrate;Ibuprofen | N02<br>M03<br>N02 |
| 9 | Trisomy 21 | Chromosomal disorder | Gabapentin<br>Metaxalone<br>Hydrocodone bitartrate;Ibuprofen | N02<br>M03<br>N02 |
| 10 | Multiple congenital abnormalities<br>Microtia | Other malformation<br>Nervous system congenital anomalies | Lamotrigine | N03 |
| 11 | Microtia | Nervous system congenital anomalies | Lamotrigine | N03 |
| 12 | Microtia | Nervous system congenital anomalies | Lamotrigine | N03 |
| 13 | Microtia | Nervous system congenital anomalies | Lamotrigine | N03 |
| 14 | Cryptorchism | Genital congenital anomalies | Topiramate<br>Folic acid | N03<br>B03 |
| 15 | Cryptorchism | Genital congenital anomalies | Topiramate | N03 |
| 16 | Spina bifida | Nervous system congenital anomalies | Levothyroxine sodium<br>Tramadol hydrochloride<br>Nabilone<br>Clonidine | H03<br>N02<br>A04<br>C02 |
| 17 | Multiple congenital abnormalities<br>Hydrocephalus<br>Cleft lip and palate<br>Double outlet right ventricle<br>Ureterocele | Other malformation<br>Nervous system congenital anomalies<br>Oro-facial clefts<br>Congenital heart disease<br>Urinary congenital anomalies | Duloxetine hydrochloride<br>Sumatriptan<br>Doxylamine succinate;Pyridoxine hydrochloride<br>Azithromycin | N06<br>N02<br>R06<br>J01 |

| | | | | |
|---|---|---|---|---|
| 18 | Atrial septal defect | Congenital heart disease | Paroxetine<br>Citalopram<br>Clomipramine<br>Cyamemazine<br>Prazepam<br>Oxazepam<br>Olmesartan<br>Etamsilate | N06<br>N06<br>N06<br>N05<br>N05<br>N05<br>C09<br>B02 |
| 19 | Cytogenetic abnormality<br>Trisomy 21 | Chromosomal disorder | Hydroxychloroquine<br>Fluoxetine<br>Duloxetine | P01<br>N06<br>N06 |
| 20 | Cytogenetic abnormality<br>Heart disease congenital<br>Dextrocardia<br>Emanuel syndrome | Chromosomal disorder<br>Congenital heart disease | Citalopram<br>Delorazepam<br>Hydroxyzine | N06<br>N05<br>N05 |
| 21 | Heart disease congenital<br>Coarctation of the aorta<br>Aorta hypoplasia<br>Ventricular septal defect<br>Cardiac aneurysm | Congenital heart disease | Carbamazepine<br>Ketoprofen<br>Paracetamol<br>Codeine<br>Tramadol<br>Botulinum toxin nos<br>Amitriptyline<br>Diazepam | N03<br>M02<br>N02<br>R05<br>N02<br>M03<br>N06<br>N05 |
| 22 | Foetal malformation | Other malformation | Emtricitabine<br>Tenofovir<br>Lopinavir<br>Ritonavir<br>Lamivudine<br>Zidovudine | J05<br>J05<br>J05<br>J05<br>J05<br>J05 |

| | | | | |
|---|---|---|---|---|
| 23 | Congenital anomaly<br>Hydrocephalus<br>Atrial septal defect<br>Cerebral ventricle dilatation<br>Congenital musculoskeletal disorder of limbs | Other malformation<br>Nervous system congenital anomalies<br>Congenital heart disease<br>Limb congenital anomalies | Naltrexone<br>Acamprosate<br>Hydrochlorothiazide<br>Irbesartan<br>Atorvastatin<br>Nicardipine<br>Ethanol | N07<br>N07<br>C03<br>C09<br>C10<br>C08<br>D08 |
| 24 | Congenital anomaly<br>Haemangioma congenital | Other malformation<br>Skin congenital anomalies | Agomelatine<br>Alprazolam<br>Oxazepam<br>Diazepam<br>Levothyroxine | N06<br>N05<br>N05<br>N05<br>H03 |
| 25 | Congenital anomaly<br>Dysmorphism<br>Clinodactyly<br>Cerebral ventricle dilatation | Other malformation<br>Limb congenital anomalies<br>Nervous system congenital anomalies | Zolpidem<br>Alprazolam<br>Tetrazepam<br>Amitriptyline<br>Atenolol<br>Nicardipine | N05<br>N05<br>M03<br>N06<br>C07<br>C08 |
| 26 | Congenital anomaly<br>Hydranencephaly | Other malformation<br>Nervous system congenital anomalies | Paracetamol<br>Codeine<br>Tramadol<br>Diclofenac | N02<br>R05<br>N02<br>M01 |
| 27 | Ventricular septal defect | Congenital heart disease | Mirtazapine<br>Zopiclone<br>Lorazepam | N06<br>N05<br>N05 |
| 28 | Congenital anomaly | Other malformation | Phenobarbital<br>Topiramate | N03<br>N03 |
| 29 | Congenital anomaly<br>Arachnodactyly | Other malformation<br>Limb congenital anomalies | Propranolol<br>Tramadol<br>Morphine | C07<br>N02<br>N02 |
| 30 | Congenital diaphragmatic hernia | Other malformation | Carbamazepine | N03 |

| | | | | |
|---|---|---|---|---|
| 31 | Congenital diaphragmatic hernia | Other malformation | Carbamazepine | N03 |
| 32 | Nasopharyngeal atresia | Oro-facial clefts | Carbamazepine | N03 |
| 33 | Choanal atresia | Respiratory congenital anomalies | Carbamazepine | N03 |
| 34 | Congenital renal disorder | Renal congenital anomalies | Carbamazepine<br>Topiramate | N03<br>N03 |
| 35 | Atrial septal defect | Congenital heart disease | Betamethasone | H02 |
| 36 | Congenital aortic valve stenosis<br>Congenital aortic valve incompetence<br>Atrial septal defect | Congenital heart disease | Fluoxetine hydrochloride<br>Omeprazole<br>Metamizole sodium<br>Insulin | N06<br>A02<br>N02<br>A10 |
| 37 | Atrial septal defect<br>Patent ductus arteriosus | Congenital heart disease | Venlafaxine hydrochloride<br>Folic acid | N06<br>B03 |
| 38 | Congenital tracheomalacia | Respiratory congenital anomalies | Venlafaxine hydrochloride | N06 |
| 39 | Pulmonary artery stenosis congenital | Congenital heart disease | Prothipendyl hydrochloride<br>Lorazepam<br>Citalopram<br>Amitriptyline hydrochloride | N05<br>N05<br>N06<br>N06 |
| 40 | Univentricular heart<br>Aorta hypoplasia | Congenital heart disease | Venlafaxine | N06 |
| 41 | Patent ductus arteriosus | Congenital heart disease | Paracetamol | N02 |
| 42 | Hypospadias | Genital congenital anomalies | Lorazepam<br>Prothipendyl hydrochloride<br>Escitalopram<br>Metamizole | N05<br>N05<br>N06<br>N02 |
| 43 | Atrial septal defect | Congenital heart disease | Betamethasone | H02 |

| | | | | |
|---|---|---|---|---|
| 44 | Cryptorchism<br>Renal aplasia | Genital congenital anomalies<br>Renal congenital anomalies | Mirtazapine<br>Duloxetine<br>Levocetirizine dihydrochloride<br>Beclometasone dipropionate<br>Salbutamol<br>Folic acid<br>Escitalopram | N06<br>N06<br>R06<br>R03<br>R03<br>B03<br>N06 |
| 45 | Atrial septal defect | Congenital heart disease | Mesalazine<br>Pantoprazole<br>Brivudine<br>Prednisolone | A07<br>A02<br>J05<br>H02 |
| 46 | Naevus flammeus<br>Telangiectasia congenital | Skin congenital anomalies | Duloxetine<br>Diphtheria vaccine toxoid;Pertussis vaccine acellular 3-component;Tetanus vaccine toxoid<br>Influenza vaccine inact split 4v<br>Amitriptyline hydrochloride | N06<br>J07<br>J07<br>N06 |
| 47 | Cryptorchism<br>Atrial septal defect<br>Ventricular septal defect<br>Congenital inguinal hernia<br>Renal aplasia | Genital congenital anomalies<br>Congenital heart disease<br>Gastro-intestinal congenital anomalies<br>Renal congenital anomalies | Duloxetine<br>Tozinameran | N06<br>J07 |
| 48 | Congenital cerebral cyst | Nervous system congenital anomalies | Levetiracetam | N03 |
| 49 | Otospondylomegaepiphyseal dysplasia | Other malformation | Oxycodone hydrochloride<br>Paracetamol | N02<br>N02 |
| 50 | Adactyly | Limb congenital anomalies | Levetiracetam<br>Oxcarbazepine | N03<br>N03 |
| 51 | Hypospadias | Genital congenital anomalies | Lormetazepam | N05 |
| 52 | Congenital anomaly | Other malformation | Milnacipran hydrochloride<br>Hydroxyzine hydrochloride | N06<br>N05 |

| | | | | |
|---|---|---|---|---|
| 53 | Cleft palate | Oro-facial clefts | Gabapentin<br>Natalizumab<br>Ebastine<br>Desloratadine | N02<br>L04<br>R06<br>R06 |
| 54 | Anterior displaced anus | Gastro-intestinal congenital anomalies | Cocaine<br>Cannabis sativa<br>Midomafetamine | N01<br>A04<br>not available |
| 55 | Congenital musculoskeletal disorder<br>Atrial septal defect | Limb congenital anomalies<br>Congenital heart disease | Etamsilate<br>Paroxetine hydrochloride<br>Olmesartan medoxomil<br>Cyamemazine<br>Prazepam<br>Citalopram | B02<br>N06<br>C09<br>N05<br>N05<br>N06 |
| 56 | Dysmorphism<br>Clinodactyly | Other malformation<br>Limb congenital anomalies | Alprazolam<br>Tetrazepam<br>Amitriptyline hydrochloride<br>Zolpidem tartrate<br>Atenolol<br>Nicardipine hydrochloride | N05<br>M03<br>N06<br>N05<br>C07<br>C08 |
| 57 | Kidney malformation | Renal congenital anomalies | Tramadol hydrochloride<br>Acetylsalicylate lysine | N02<br>N02 |
| 58 | Foetal malformation<br>Limb reduction defect<br>Congenital scoliosis<br>Single functional kidney | Other malformation<br>Limb congenital anomalies<br>Skeletal congenital anomalies<br>Renal congenital anomalies | Diazepam<br>Olanzapine<br>Buprenorphine hydrochloride<br>Zopiclone<br>Omeprazole | N05<br>N05<br>N02<br>N05<br>A02 |
| 59 | Congenital musculoskeletal disorder | Limb congenital anomalies | Quetiapine<br>Sertraline<br>Buprenorphine hydrochloride<br>Zopiclone | N05<br>N06<br>N02<br>N05 |

| | | | | |
|---|---|---|---|---|
| 60 | Horner's syndrome | Nervous system congenital anomalies | Folic acid<br>Paracetamol | B03<br>N02 |
| 61 | Spina bifida | Nervous system congenital anomalies | Folic acid | B03 |
| 62 | Congenital anomaly<br>Ventricular septal defect | Other malformation<br>Congenital heart disease | Gabapentin | N02 |
| 63 | Congenital anomaly<br>Ventricular septal defect | Other malformation<br>Congenital heart disease | Gabapentin | N02 |
| 64 | Ventricular septal defect | Congenital heart disease | Gabapentin | N02 |
| 65 | Abdominal wall anomaly<br>Spina bifida<br>Ectopia cordis | Abdominal wall defects<br>Nervous system congenital anomalies<br>Congenital heart disease | Paroxetine<br>Methylphenidate<br>Haloperidol<br>Zopiclone | N06<br>N06<br>N05<br>N05 |
| 66 | Heart disease congenital | Congenital heart disease | Amitriptyline<br>Folic acid | N06<br>B03 |
| 67 | Multiple congenital abnormalities | Other malformation | Temazepam<br>Clobazam<br>Diazepam | N05<br>N05<br>N05 |
| 68 | Ventricular septal defect | Congenital heart disease | Colchicine | M04 |
| 69 | Heart disease congenital | Congenital heart disease | - | - |
| 70 | Meningomyelocele | Nervous system congenital anomalies | - | - |
| 71 | Congenital anomaly | Other malformation | - | - |
| 72 | Cleft lip<br>Cleft palate<br>Congenital foot malformation | Oro-facial clefts<br>Limb congenital anomalies | - | - |
| 73 | Cytogenetic abnormality<br>Trisomy 21 | Chromosomal disorder | - | - |
| 74 | Congenital anomaly<br>Limb reduction defect | Other malformation<br>Limb congenital anomalies | - | - |
| 75 | Cytogenetic abnormality<br>Trisomy 21 | Chromosomal disorder | - | - |

| | | | | |
|---|---|---|---|---|
| 76 | Pyloric stenosis | Gastro-intestinal congenital anomalies | - | - |
| 77 | Hemivertebra | Skeletal congenital anomalies | - | - |
| 78 | Ventricular septal defect | Congenital heart disease | - | - |
| 79 | Ventricular septal defect | Congenital heart disease | - | - |
| 80 | Ventricular septal defect | Congenital heart disease | - | - |
| 81 | Ventricular septal defect | Congenital heart disease | - | - |
| 82 | Ventricular septal defect | Congenital heart disease | - | - |

*Abbreviations*: ICSR, individual case safety report; ATC, anatomical therapeutic chemical
